# Serological evidence of Zika virus infection in febrile patients at Greater Accra Regional Hospital, Accra Ghana

**DOI:** 10.1186/s13104-019-4371-4

**Published:** 2019-06-10

**Authors:** Godson Aryee Ankrah, Joseph Humphrey Kofi Bonney, Esinam Eudosia Agbosu, Deborah Pratt, Theophilus Korku Adiku

**Affiliations:** 1 Department of Medical Microbiology, College of Health Sciences, University of Ghana, Korle Bu, Accra Ghana; 2 Virology Department, Noguchi Memorial Institute for Medical Research, College of Health Sciences, University of Ghana, P.O. Box LG 581, Legon, Accra Ghana; 3 Department of Biomedical Sciences, University of Health and Allied Sciences, Ho, Volta Region Ghana

**Keywords:** Zika virus, Seroprevalence, Anti-Zika virus immunoglobulins M and G (IgM and IgG) antibodies, Enzyme linked immunosorbent assay (ELISA)

## Abstract

**Objective:**

Increase in the evidence of global occurrence of Zika viral infection suggests that in Africa the circulation of the virus which causes 80% of asymptomatic infection could be undetected and/or overlooked. We sought to serologically detect Zika virus infection in febrile patients at Greater Accra Regional Hospital, Ghana.

**Results:**

Of the 160 patient serum samples analyzed, 33 were found to have antibodies against Zika virus infection. Among the sero-positives 30 (91%) of the cases were anti-Zika virus IgM with the 21–30-year age group recording the highest number of 8 (26%) and 2 (7%) cases being the least for the 61 years and above age group. All sero-positive febrile patients developed at least one symptom consistent with Zika virus infection: 33 (100%) fever, 25 (76%) muscle pain, 24 (73%) joint pain, and conjunctivitis 2 (6%). Digestive symptoms recorded include 16 (49%) nausea, 12 (36%) vomiting and diarrhea 18 (55%). In addition, 28 (85%) loss of appetite, 14 (75%) rapid respiration and chest pain 15 (42%) were reported by seropositive febrile patients. Our data indicates exposure to Zika virus which suggests the possible circulation of the virus among febrile patients in Ghana with a sero-prevalence rate of 20.6%.

## Introduction

Febrile illnesses account for about 30% and 75% of healthcare visit by children [1] and adults [2] respectively in Ghana. However, these febrile illnesses are mostly misdiagnosed for endemic diseases, malaria and typhoid fever. Zika virus infection shares overlapping signs and symptoms with most endemic diseases including malaria and typhoid fever. The etiologic agent, Zika virus was first isolated in Uganda in 1947 from a rhesus macaque monkey and *Aedes africanus* mosquito in 1948 [3]. First human cases of Zika virus infection were reported in Africa and Asia. In Africa, Zika virus infection was documented in Uganda and Tanzania in 1952 [4] and in Asia, notably Indonesia, Malaysia and Thailand [5]. Afterwards serological studies detected Zika virus infection in humans in Nigeria, Egypt, India, Pakistan, North Vietnam and Philippines, spreading from Africa to Southeast Asia [6, 7]. Zika virus infection in Africa and Asia never got the deserved attention due to the sporadic nature of the infection coupled with mild short-term and low-grade fever (37.8–38.5 °C) and mostly self-limiting symptoms including maculopapular and pruritic rashes, conjunctivitis (non-purulent) and arthralgia [8]. As a result, Zika virus infection appeared to have been neglected in tropical medicine and no efforts were made to develop vaccine and drugs for treatment [7]. Although Zika virus infection has not been reported in Ghana, evidence of several outbreaks and exposure to the virus have been reported in various African countries such as Gabon [9] Cape Verde, Senegal [10, 11] and Ivory Coast which share a common border with Ghana. Thus, the evidence of exposure to the virus in our neighboring countries and the presence of active vector population of *Aedes* spp. of mosquitoes in Ghana have necessitated the need to document the exposure levels in febrile patients. The mild or asymptomatic nature and the overlapping clinical feature with other endemic disease conditions make it probable for typical Zika virus infection to be missed out in diagnosis. Therefore, we set out to augment and improve scientific data in the sub-region and create awareness to pre-empt possible outbreaks with the detection of antibodies against Zika virus circulating among febrile patients at Greater Accra Regional Hospital, Accra Ghana.

## Main text

### Methods

#### Study design

This was a cross sectional study that involved 160 archived serum samples from febrile patients at the Greater Accra Regional Hospital between December 2016 and November 2017. These clinical specimens were obtained as part of an ongoing project with the aim of using serological and molecular tools to detect Dengue, Chikungunya and other Arboviruses in febrile patients within selected health facilities in Ghana. A structured case investigation form was used to collect information about demographic features, and clinical signs and symptoms of the febrile patients.

#### Eligibility criteria

Inclusion criteria for enrollment included a person with fever (body temperature above 38 °C) and at least one of the following signs or symptoms: arthralgia; or arthritis; or conjunctivitis (non-purulent/hyperemic). Patients positive for malaria by blood film diagnosis and patients who refuse to submit samples after consenting were excluded from the study. Venous blood was collected into heparinized tubes and centrifuged, and serum was harvested, aliquoted into two separate cryogenic 1.8 ml vials for each patient sample and stored at − 80 °C.

#### Laboratory testing

The archived sera from febrile patients were tested by enzyme linked immunosorbent assay (ELISA) for anti-Zika virus IgM and IgG antibodies with a commercial ELISA kit, Zika virus IgM and IgG capture ELISA (Abcam, Cambridge, UK). The sensitivity of this ELISA kit used (Abcam anti-Zika virus IgM and IgG) was reportedly assessed by evaluating the performances of 5 commonly used Zika virus immunoassays against an ELISA (MAC ELISA) developed by the US Centers for Disease Control and Prevention and cross-Plaque reduction neutralization test (PRNT) was reported as 57% and a specificity of 100% [12]. Manufacturer’s instructions or recommendations were followed in all tests performed.

Zika virus IgM and IgG negative control, Zika virus IgM and IgG positive control and the test serum samples were first diluted with sample dilution buffer for Zika virus IgM and IgG. Thereafter, incubated at 37 °C for an hour in micro-titre plates pre-coated with monoclonal antibody bound to recombinant Zika virus antigen. After subsequent washing, wells were treated with horseradish peroxidase (HRP) Zika virus conjugate (peroxidase labeled Zika virus antigen) except for the substrate blank well and incubated for 30 min at 37 °C. Washing was repeated after which substrate solution 3,3′,5,5′-tetramethylbenzidine (TBM) hydrogen peroxide was added and incubated in the dark at room temperature 20–25 °C for 15 min. A stop solution was then added, and the absorbance measured at 450 nm using ELISA microtiter plate reader (Human Diagnostic Worldwide, Germany) within 30 min after terminating the reaction using a reference wavelength of 620 nm. The ELISA tests results were classified according to the interpretation provided by the supplier, Abcam, Cambridge, UK.

#### Statistical analysis

One Way-Anova was used to test the association within groups at significance level of P-value < 0.05 using SPSS version 20.

### Results

#### Characteristics of Zika virus sero-positives

Of the 160 samples tested, 33 (20.6%) had positive serological evidence for Zika virus. Out of the 33 Zika virus sero-positives, 30 (90.9%) were IgM positive whereas 3 (9.1%) were IgG positive. Of all the patients enrolled, 119 (74.4%) were females while 41 (25.6%) were males. Number of anti-Zika virus IgM males was 11 (36.7%) and that of females was 19 (63.3%) (Table 1). Anti-Zika virus IgG antibodies were detected only in females, however Zika virus infection was observed to affect males and females in a ratio of 1:2. Among the age groups identified, 8 (26.7%) of the anti-Zika virus IgM were recorded in age group 21–30 with 2 (6.7%) for the age group 61 years and above (Table 1). Anti-Zika virus IgG antibodies were detected only in age groups 21–30 2 (66.7%) and 41–50 1 (33.4%) (Table 1). There was significant difference between anti-Zika virus IgM among the age groups (P = 0.004).

**Table 1:** Demographic distribution of seropositive febrile patients

Variables	Zika virus antibodies, n = 33			
	IgM (30)	*P*-value	IgG (3)	*P*-value
Age groups				
3–20	4 (13%)	0		
21–30	8 (26.7%)	2 (66.7%)		
31–40	6 (20%)		0	
41–50	7 (23.3%)	0.004*	1 (33.3)	0.203
51–60	3 (10%)		0	
61 and above	2 (6.7%)	0		
Gender				
Male	11 (36.7%)		0	
Female	19 (63.3%)	0.166	3 (100%)	0.500

*n* number of Zika virus seropositive

* Statistically significant difference between age groups (P = 0.004). P is significant at 0.05

#### Monthly distribution of Zika virus antibodies

**Fig. 1:**
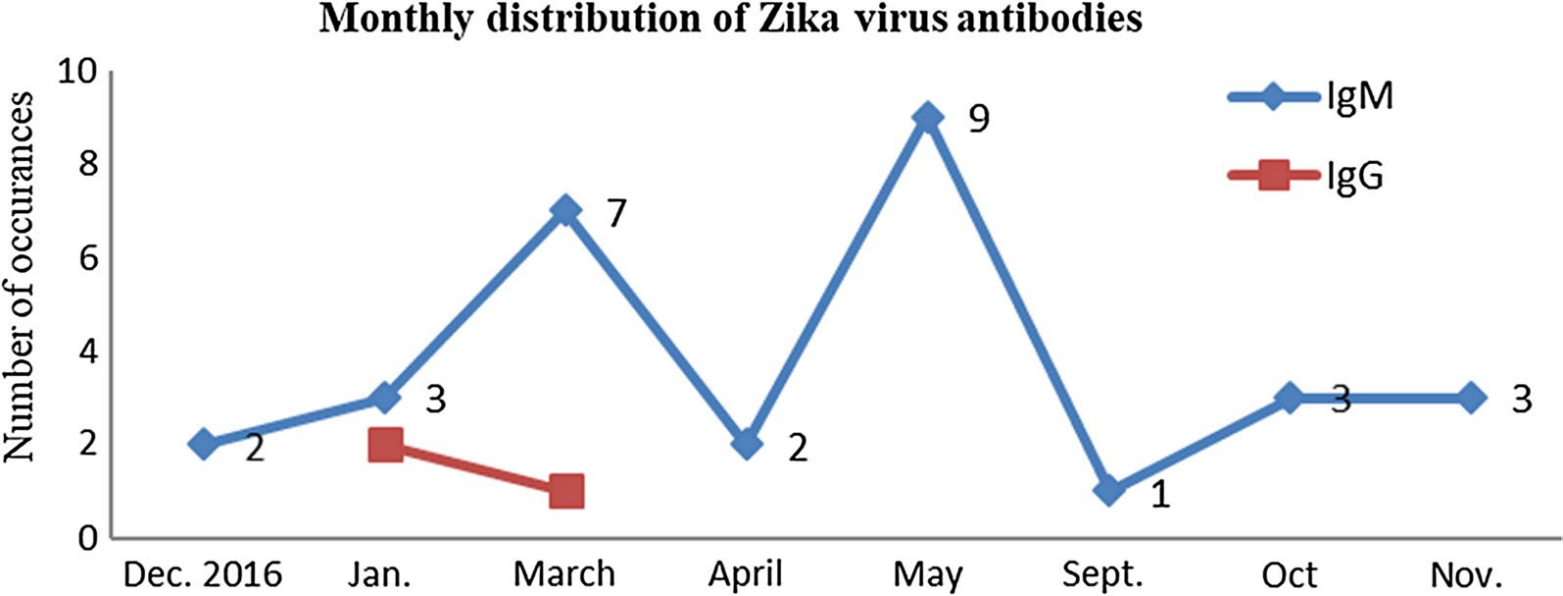
Monthly distribution of Zika virus antibodies

Anti-Zika virus IgM detection peaked in March 7 (23.3%) and May 9 (30%) and lowed in September 1 (3.3%). However, anti-Zika virus IgG antibodies were detected only in January and March 2 (66.7%) and 1 (33.3%) respectively (Fig. 1).

#### Symptoms presented by seropositive febrile patients

Thirty-three (100%) of the seropositive febrile patients were presented with fever. The least 1 (0.8%) symptom reported was conjunctivitis which was also not reported by any seropositive Zika virus female. All other symptoms were reported in both males and females (Table 2).

**Table 2:** Symptoms and complications of Zika virus seropositives (n = 33)

Symptoms	Zika positive cases	Percentage
Fever	33	100
Diarrhea	18	55
Nausea	16	49
Vomiting	12	36
Loss of appetite	28	85
Muscle pain	25	76
Joint pain	24	73
Conjunctivitis	2	6
Chest pain	15	46
Rapid respiration	14	42
Jaundice	1	3
Recent hearing loss	1	3

### Discussion

The results from the study indicate exposure levels to Zika virus which suggest the possible circulation of the virus among febrile patients in Ghana with a sero-prevalence of 20.6%. Anti-Zika virus IgM prevalence observed is likely due to the recent (in the year 2016) wave of Zika virus infection dominated headlines in certain parts of the world, especially those with tropical climate. Data presented by this study again suggests noticeable levels of susceptibility to Zika virus infections. This could mean many infected women subjects being at risk of giving birth to babies with defects. Thus, an extended scope to cover infected expectant mothers and diagnose birth defects would have been interesting and will be considered in further studies.

The sero-prevalence of Zika virus antibodies of 20.6% in this study was consistent with study findings from other African countries with a rate from as low as 0.1% in Gabon and Senegal and as high as 38% in Cameroon [13]. These differences could be due to inconsistency in the study participants’ inclusion criteria or diagnostic test used. Zika virus infection was estimated to affect females and males in a ratio of 2:1 which correlate well with several studies [14]. Considering the findings of [14] and that of this study, there may be gender-related differences in Zika virus infection incidence, which might be due to exposure differences [15]. Activeness of females during the early hours of the day exposes greater proportion of females to Zika virus-carrying *Aedes* spp. of mosquitoes either at work or while travelling to and from work. This may also be attributed to possible differences in who sought medical care following symptomatic Zika virus infection. High prevalence of IgM or IgG in age group 21–30 compared to 61 and above support data from earlier studies by [14] and [16]. Possible explanation to this observation could be the activeness of age group 21–30 during the early hours of the day which exposes them to the bites of Zika virus-carrying *Aedes* spp. of mosquitoes more frequently than age group 61 years and above. This could also suggest adults manifest with disease less, as they become immune to Zika virus. The proportions of the IgG antibody titers recorded in age group 21–30 and 41–50 may suggest that, Zika virus infection is not endemic in Ghana, rather the virus had been introduced to a non-exposed population as endemicity is attained when the adult infection decreases and only the new entrants into the population are affected more [16]. The significant difference between IgM among the age groups may be due to the pattern of erotic activities and exposure among the age groups. Active sexual activity and exposure begins early, increases simultaneously and declines as one ages. Monthly distribution of Zika virus infection with higher prevalence in the raining season as seen in this study is in harmony with reported pattern of Zika virus transmission [17]. The high frequency of IgM in the month of May and March could be due to the high amount of rainfall which provided temperatures suitable for virus survival, incubation, development and biting of Aedes aegypti and Aedes albopictus [18]. The compact clusters of cases in March and May, and the high prevalence of IgM against Zika virus are consistent with an acute outbreak of Zika virus infection in the population without previous immunity to Zika virus [19]. In this study, commonly reported symptoms of Zika virus infection documented were fever, muscle pain, joint pain and conjunctivitis. Strikingly, conjunctivitis often observed in Zika virus infection was not reported by any seropositive female. Digestive symptoms noted in Zika virus infected patients but rarely observed [20], however, found in this study were nausea, vomiting and diarrhea.

## Limitation

The limitation of this study was the absence of plaque neutralization reduction test and PCR for confirmation.

## Data Availability

In this manuscript, the availability of data and material was not applicable.

## Abbreviations

AMED: Japan Agency for Medical Research and Development
ELISA: enzyme-linked immunosorbent assay
HRP: horseradish peroxidase
IgG/M: immunoglobulin G/M
J-GRID: Japan Initiative for Global Research Network on Infectious Diseases
NMIMR: Noguchi Memorial Institute for Medical Research
TBM: 3,3′,5,5′-tetramethylbenzidine
US CDC: United States of America’s Centers for Disease Control and Prevention
